# The Lignicolous Fungus *Hericium erinaceus* (Lion's Mane Mushroom): A Promising Natural Source of Antiradical and DPPH Inhibitory Agents

**DOI:** 10.1155/tswj/5964432

**Published:** 2025-02-03

**Authors:** Akbar Jahedi, Saeideh Ahmadifar, Rahman Mohammadi, Ebrahim Mohammadi Goltapeh

**Affiliations:** ^1^Department of Plant Pathology, Faculty of Agriculture, Tarbiat Modares University, Jalal Al Ahmad Street, No. 7, Tehran, Iran; ^2^Department of Forest Biology, Faculty of Natural Resources, Tarbiat Modares University, Jalal Al Ahmad Street, No. 7, Tehran, Iran; ^3^Department of Entomology, Faculty of Agriculture, Tarbiat Modares University, Jalal Al Ahmad Street, No. 7, Tehran, Iran

**Keywords:** agricultural wastes, antioxidant, *Hericium erinaceus*, PCA, waste management

## Abstract

Nowadays, the importance of the genus *Hericium* is increasing due to its nutraceutical and pharmaceutical properties. The main idea of this study is to ingenious management of these wastes to achieve the highest performance of nutrients, minerals, and antioxidant properties using enriched agricultural waste. After preparing mushroom samples, the amount of mineral and mycochemical substances have been respectively evaluated by the Association of Official Analytical Chemists and Folin–Ciocalteu assay. In conclusion, among the fruiting bodies' samples harvested from 19 substrates, the substrates sawdust 30% + wheat straw 30% + bagasse 15% + rice bran 15% + corn flour 10% recorded the highest phenol and flavonoid contents, with values of 27 mg GAE/g DW ext. and 8 mg QE/g DW ext. as well as the IC50 (88.7 μg/mL). P and K's highest amounts were recorded with the values of 1833 and 1600 mg/100 g DW, and Fe was recorded with values of 77.7 mg/100 g DW. This study, therefore, provides understandings on the biological technologies for the valorization of organic solid waste into valuable and useful bio-based products.

## 1. Introduction

In recent years, rapid developments in the agricultural food sector due to rapid growth of population worldwide lead to generating a significant amount of lignocellulosic waste year by year [1]. However, many of these waste materials can be revived, and thus they can become a resource for energy generation or industrial production, if managed intelligently. One of the proper approaches for the optimal usage of agroindustrial wastes is using their biological capacity. Agrowaste substrates can be used as a substrate for the production of medicinal-edible mushroom due to complex lignocellulosic compounds being broken down [2], and its positive effects have been reported to increase mushroom production, yield and biological efficiency, and chemical properties of different fruiting bodies of mushrooms [3]. For the past decade, the nutrient and bioactive substances of mushrooms, as a novel alternative food raw material, have attracted the attention of many researchers and consumers [4]. *Hericium erinaceus* is a vital edible medicinal mushroom worldwide, especially in Asia. *H. erinaceus* is commonly known as the “Yamabushitake” in Japan and the “monkey head” in China [5]. Tan et al. [6] stated that 100 g dried weight of *H. erinaceus* fruit body contains 44.9 g of carbohydrate, 26.3 g of protein, 6.4 g of crude fiber, 4.2 g of fat, and 10.2 g of water. In addition, including the mineral amounts of phosphor (P) 85 mg, calcium (Ca) 2 mg, iron (Fe) 18 mg, thiamine (0.69 mg), riboflavin (1.89 mg), and carotene (0.01 mg) [6], *H. erinaceus* has long been regarded as a major source of valuable bioactive ingredients due to its useful bioactive metabolites, such as steroids, phenolic compounds, alkaloids, erinacines, essential amino acids, monounsaturated fatty acids, polyunsaturated fatty acids, glycoproteins, and polysaccharides [7]. Many bioactive compound types from *H. erinaceus* have appropriate therapeutic and pharmacological properties [8]. According to the results of Li et al. [9], in the polysaccharides of *H. erinaceus*, β-glucan content was higher than others. Some mushroom species that belong to the genus *Hericium* have been widely recorded to have different pharmacological properties, such as anticancer, immunomodulatory, anti-inflammatory, antimicrobial, antioxidant, and liver protection activities [9–14]. According to the results of Wang et al. [15], *Hericium* spp. fungus is caused by a reduction in blood glucose levels, the regulation of blood lipid levels, and antioxidant activities. Moreover, Son et al. [16] also reported that the *H. erinaceus* mushroom is a pharmaceutical mushroom with anticancer and antimicrobial activities that can be found throughout almost the entire Northern Hemisphere, China, Japan, Russia, and Europe, except in tropical and polar regions [17]. Some varieties of *H. erinaceus* have higher phenolic and antioxidant contents but lower moisture contents [18–20]. In summary, the aims of the current study include (i) providing a brief overview of the compound and pharmaceutical properties of *H. erinaceus,* (ii) determining the mineral element concentrations (potassium [K], Ca, copper [Cu], P, magnesium [Mg], manganese [Mn], zinc [Zn], and Fe), and (iii) quantify the content of phenolic compounds and antioxidant activity of native mushrooms from the Hyrcanian region of Iran.

## 2. Materials and Methods

### 2.1. Lion's Mane Sample Collection and Preparation

Dry fruiting body samples of Lion's mane cultivated on 19 different substrates were prepared from the Department of Plant Pathology, Faculty of Agriculture, University of Tarbiat Modares, Tehran, Iran. The germplasm resources of the samples were preserved in the laboratory of the plant pathology department, agricultural faculty of Tarbiat Modares University, Tehran, Iran. For content analyses, all 19 samples were completely dried and powdered. All steps during milling were performed carefully to avoid any cross-contamination. All tools and equipment were autoclaved with 0.1% diethyl pyrocarbonate solution at 37°C overnight, at 121°C for 20 min, and then dried at 100°C before use. Powdered Lion's mane samples were transferred into sterilized containers and kept at 4°C for further tests [21].

### 2.2. Mineral Content of *H. erinaceus*

A sample of dried samples of *H. erinaceus* sporophores grown on 19 defined substrates from local agroindustrial wastes was subjected to mineral analysis. This test was performed based on the Association of Official Analytical Chemists [22]. Five hundred mg of each sample was subjected to dry-ash mineralization in a furnace at 450°C. The incineration residue was extracted with 0.5 mL/mL HNO3 and 0.5 mL/mL HCl and made into an appropriate volume of distilled water into which Zn, Mn, Cu, and Fe were weighed directly. The other elements were specified after dilution [23]. All measurements were done in air/acetylene flame atomic absorption spectroscopy (AAS) on an Analyst 200 Perkin Elmer equipment (Perkin Elmer, Waltham, MA, USA), comparing absorbance responses with purity analytical standard solutions for AAS made with Fe (NO_3_)_3_, Cu (NO_3_)_2_, Mn (NO3)2, Zn (NO_3_)_2_, and Mg band, supplied by Merck (Darmstadt, Germany) and Panreac Quí-mica (Barcelona, Spain).

### 2.3. Phytochemical Content of *H. erinaceus*

To measure the amount of total phenol, the Folin–Ciocalteu method was applied [20]. For this purpose, extracts of samples were prepared with a concentration of 0.01 g/mL methanol. In addition, seven percent sodium carbonate dissolved in distilled water was prepared separately. Then, 20 μL of the extract was poured into the tube, and two mL of distilled water and 100 μL of Folin were added to it. After 30 min after the addition of Folin, 300 μL of sodium carbonate was also added. All treatments were prepared in three replicates. Then, the obtained solution was placed in a shaker incubator for two hours, and finally, it was determined by a spectrophotometer at 765 nm. Total phenolic content (TPC) was considered in mg of gallic acid equivalent per gram of dry weight extract (mg GAE/g DW ext) by the linear equation obtained from the gallic acid standard calibration curve [24]. The flavonoid content was determined based on the aluminum chloride method described by Slinkard and Singleton [25]. First, extracts were prepared with a concentration of 500 mg/L methanol. In addition, an amount of two percent aluminum chloride dissolved in methanol was prepared. Then, 600 microliters of the extract were collected in a test tube, 600 μL of two percent aluminum chloride was added, and the samples were prepared in three replicates. After 10 min, the resulting solution was determined by a spectrophotometer at 420 nm. Total flavonoid content (TFC) was considered as quercetin equivalent milligrams per dry matter gram of the mushroom extract (mg QE/g DW).

### 2.4. Antioxidant Activity

To determine the antioxidant activity, the radical scavenging activities (RSAs) of the dried mushroom extracts were estimated using a 2,2-diphenyl-1-picrylhydrazyl (DPPH) assay based on the method by Shimada et al. [26]. First, the methanol extract from mushrooms was prepared with concentrations of 1000, 3000, 10,000, and 300 mg/L methanol. In this step, DPPH was prepared with a concentration of 400 mg/L of methanol. Then, a mixture with a volume of 200 microliters containing DPPH and methanol in a ratio of 1:1 and 30 microliters of various concentrations (10, 30, 50, 100, and 200 μg/mL) of the extracts was prepared, and each mixture was poured in a plate of 96 wells with three repetitions. The samples were placed in a shaker incubator in the dark for one hour. The sample's absorbance was immediately measured with an ELISA Reader at 517 nm.

The percentage of free radical scavenging was measured based on the calibration curve with the formula: ([A1 − A2]/A1) × 100, where A1 is the control absorbance and A2 is the sample absorbance. Ascorbic acid was used as the positive control. In addition, to compare the activity of extracts, the IC50 parameter was used (IC50 is the extract concentration that inhibits 50% of free radicals).

### 2.5. Analysis

Data analysis was done using SAS version 9.4. Data analysis was performed using a completely randomized design. The Kolmogorov–Smirnov test was used for the normality of the data, and the homogeneity of variance was checked at the 1% level. The least significant difference (LSD) test was used to check the significant difference between the treatments. Pearson's correlation-significance matrix, cluster, and principal component analysis (PCA) were generated using SAS and Minitab 17, respectively.

## 3. Results and Discussion

### 3.1. Macro- and Microelements

In the previous research, it was reported that medicinal mushrooms contain many mineral elements [27]. The mineral content of fruit bodies is different with various substrates (Table 1). In total, the mushrooms cultivated on 19 substrate formulations were of good quality in terms of mineral content. Calcium (Ca) and potassium (K) contents were higher than those of other minerals in mushrooms. When base materials (sawdust, baggase, or wheat straw) were mixed with supplements (corn flour, rice bran, wheat bran, or soybean powder) at various rates in substrate formulas, the mineral contents of samples were improved. Among the macroelements, the highest amounts of K, P, Ca, and Mg were recorded in the substrates on sawdust 30% + wheat straw 30% + rice bran 20% + soybean powder 10% + bagasse 10% and bagasse 100%, with values of 1600, 1833, 2267, and 1463 mg/100 g DW, respectively. Among the microelements, the highest amounts of Fe, Cu, Mn, and Zn in the substrates sawdust 30% + wheat straw 30% + bagasse 15% + rice bran 15% + corn flour 10%, sawdust 30% + wheat straw 30% + wheat bran 20% + soybean powder 10% + bagasse 10%, sawdust 60% + soybean powder 40%, and sawdust 30% + wheat straw 30% + bagasse 15% + wheat bran 15% + corn flour 10% were 77.7, 1.07, 4.33, and 6.67 mg/100 g DW, respectively. The results of studies have showed that K is the main element in mushrooms [28], and this was also found in the current study. According to Crisan and Sands [29], mushrooms have large amounts of minerals that they absorb from their substrate. Therefore, the difference in minerals and the concentration of elements is highly dependent on the cultivation method and the amount of minerals in the substrate used [30]. Previous studies [31] reported that the amounts of macroelements in the substrate compositions increased with the addition of Supporting Information and agricultural waste compared to the control (Magnolia sawdust), and the decrease in the amount of macro- and microelements in the cultivated substrates during the cultivation process showed the mycelium and fruiting body of mushrooms.

### 3.2. DPPH Scavenging Activity and Phytochemical Content of *H. erinaceus*

Fungi accumulate different secondary metabolites, including steroids, polyketides, terpenes, and phenolic compounds. Among the secondary metabolites, phenolic compounds are well related to lipid peroxidation inhibition, free RSA, metal chelation, and reducing power, probably because of their hydroxyl groups [32]. Phenolic compounds including tannins, phenolic acids, and flavonoids are considered the main factors of plants' antioxidant capacity. In addition, these antioxidants have different biological activities, such as antiatherosclerotic, anti-inflammatory, and anticancer activities [33].

The TPC in the dry extract is considered in milligrams of gallic acid equivalent, and the TFC is considered in milligrams of quercetin equivalent. However, in the current study, the total phenolic yield remained almost constant with increasing fourth (100 μg/mL) and fifth (200 μg/mL) concentrations. As the extract concentration increased from 10 μg/mL to 200 μg/mL, the total phenolic yield increased. It should be noted that flavonoids are also present in the *H. erinaceus* extract, although they are present in very small amounts.

**Figure 1:**
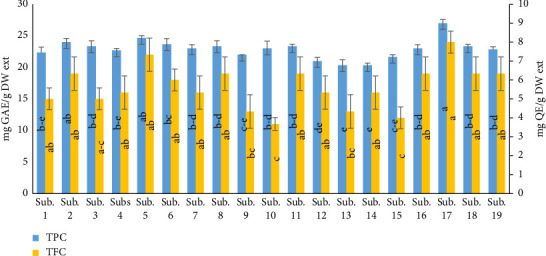
Phytochemical contents of *H. erinaceus* harvested from 19 various substrates. Values were considered as mean ± SD of triplicates. Means with various letters were significantly different (*p* < 0.01). TFC: total flavonoid content; TPC: total phenol content Sub. 1 (wheat straw 100%), Sub. 2 (sawdust 100%), Sub. 3 (bagasse 100%), Sub. 4 (wheat bran 40% + wheat straw 60%), Sub. 5 (sawdust 60% + wheat bran 40%), Sub. 6 (bagasse 60% + wheat bran 40%), Sub. 7 (wheat straw 60% + rice bran 40%), Sub. 8 (sawdust 60% + rice bran 40%), Sub. 9 (rice bran 40% + bagasse 60%), Sub. 10 (wheat straw 60% + corn flour 40%), Sub. 11 (sawdust 60% + corn flour 40%), Sub. 12 (bagasse 60% + corn flour 40%), Sub. 13 (soybean powder 40% + wheat straw 60%), Sub. 14 (sawdust 60% + soybean powder 40%), Sub. 15 (bagasse 60% + soybean powder 40%), Sub. 16 (sawdust 30% + wheat straw 30% + bagasse 15% + rice bran 15% + corn flour 10%), Sub. 17 (sawdust 30% + wheat straw 30% + bagasse 15% + wheat bran 15% + corn flour 10%), Sub. 18 (sawdust 30% + wheat straw 30% + rice bran 20% + soybean powder 10% + bagasse 10%), and Sub. 19 (sawdust 30% + wheat straw 30% + wheat bran 20% + soybean powder 10% + bagasse 10%).

According to the results of the present study, the TPC of treated beds with increasing percentages of supplements and additives compared to the control (wastes alone) was an increase, which is in accordance with the results of the authors in [34], could arise from variations in genetic backgrounds, environmental factors, and cultivation practices as well. The possible reasons for the varied results for different amounts of total phenolic and flavonoid content and antioxidant activities between the present study and the studies of other researchers may be due to different extraction conditions and differences in the presence of phenolic compounds and substrates and other secondary metabolites [35]. The extraction efficiency of total phenol strongly depends on the type of sample which is affected by the concentration of phenols and flavonoids and the type of extraction solvent used [36]. The results strongly indicate that flavonoids and phenolics are the main components of the *H. erinaceus* extracts, which can explain their high RSA. Phenolic compounds act as reducing agents, hydrogen donors, and are capable of scavenging free radicals. The phenolic (TPC) and flavonoid (TFC) contents of *H. erinaceus* were measured using the Folin–Ciocalteu reagent (Figure 1). The TFC reached 8 mg QE/g DW extract, and the TPC of *H. erinaceus* reached 27 mg GAE/g DW extract. These findings were consistent with previous findings, who reported that the differential TPC, TFC contents, and antioxidant activities from different mushroom may plausibly be due to geographical variations in chemical constituents, nature of sample, nutritional conditions, and extraction method [37].

### 3.3. Antioxidant Activity of *H. erinaceus*

**Figure 2:**
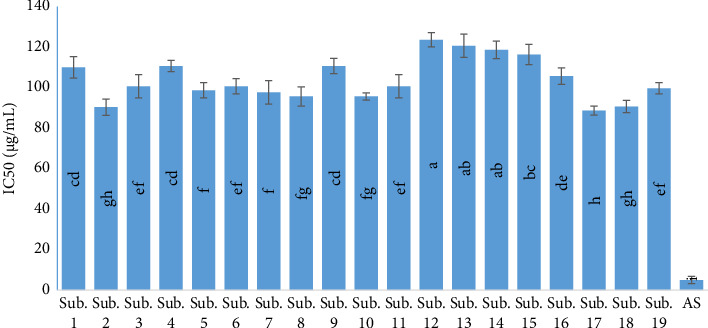
Antioxidant activity of *H. erinaceus* harvested from 19 various substrates. Values were considered as mean ± SD of triplicates. Means with various letters were significantly different (*p* < 0.01). AS: ascorbic acid as control Sub. 1 (wheat straw 100%), Sub. 2 (sawdust 100%), Sub. 3 (bagasse 100%), Sub. 4 (wheat bran 40% + wheat straw 60%), Sub. 5 (sawdust 60% + wheat bran 40%), Sub. 6 (bagasse 60% + wheat bran 40%), Sub. 7 (wheat straw 60% + rice bran 40%), Sub. 8 (sawdust 60% + rice bran 40%), Sub. 9 (rice bran 40% + bagasse 60%), Sub. 10 (wheat straw 60% + corn flour 40%), Sub. 11 (corn flour 40% + sawdust 60%), Sub. 12 (bagasse 60% + corn flour 40%), Sub. 13 (soybean powder 40% + wheat straw 60%), Sub. 14 (sawdust 60% + soybean powder 40%), Sub. 15 (soybean powder 40% + bagasse 60%), Sub. 16 (sawdust 30% + wheat straw 30% + bagasse 15% + rice bran 15% + corn flour 10%), Sub. 17 (sawdust 30% + wheat straw 30% + bagasse 15% + wheat bran 15% + corn flour 10%), Sub. 18 (sawdust 30% + wheat straw 30% +rice bran 20% + soybean powder 10% + bagasse 10%), and Sub. 19 (sawdust 30% + wheat straw 30% + wheat bran 20% + soybean powder 10% + bagasse 10%).

Antioxidant activity is closely related to flavonoids, anthocyanin, TPC, and vitamins. Of course, according to many researchers, antioxidant activity is more influenced by total phenol [38]. This is consistent with the study by several authors who stated that the water extract *Termitomyces heimii* and *T. mummi* forms were found to possess better antioxidant activities (*p* < 0.05), and a general correlation between higher antioxidant activity and a larger amount of total phenolics [39, 40]. As measured by biochemical methods, the activity of antioxidants considered by the percent inhibition of the reaction of lipid peroxidation was the highest in the extract of *H. erinaceus*. The results certified a correlation between these remarkable activities of antioxidants and a compound's high content with the properties of free radical scavenging. The results of previous research studies show that the amount of phenolic compounds is affected by factors such as variety, environmental conditions, cultivation operations, harvesting stage, and drying and storage conditions. Among the environmental factors affecting the accumulation of phenolic compounds are light intensity and temperature [41]. Therefore, it can be concluded that the high content of total phenol and flavonoids and of course the high antioxidant properties of the mushrooms harvested from these substrates are due to the high content of the above compounds in their growth medium. As shown in Figure 2, the maximum activity of antioxidants of *H. erinaceus* related to an IC50 of 88.67 μg/mL. According to Bao et al. [42], the IC50 of *H. erinaceus* mycelia cultivated using the submerged method was 13.67 mg/mL, while Aramsirirujiwet and Kimkong [43] stated that the IC50 of *H. erinaceus* mycelia ranged from 1.26 to 3.15 mg/mL.

Based on the study by Zhai et al. [44], the *Agaricus* spp. cultivation of wheat bran can increase the antioxidant and phenolic content activities of the mycelia, while the cultivation of *A. blazei* and *Cordyceps sinensis* on rice grain can increase the chemical composition and nutritional value of mycelia [42, 45]. In addition, the bag cultivation of *Inonotus obliquus* on mulberry extract and corn grain can enrich the bioactive compounds and antioxidant activity level of the fruit body, while the cultivation of *H. erinaceus* along with corn powder improves the nutritional value of the harvested fruiting bodies [46].

DPPH RSA is strongly related to the sample concentration. In general, DPPH RSA increases with increasing sample concentration. According to Sun and Ho [47], the yield of the extract depends on the solvent type, temperature, extraction time, and the chemical nature of the sample. The chemical characteristics and the solvent used for the samples are two main factors. In addition, the extraction method used is effective in antioxidant activity [47].

In general, the results of this study revealed that the inhibition of DPPH free radicals increased with increasing concentration. At a concentration of 200 μg/mL, the highest inhibition of DPPH was observed in the substrates sawdust 30% + wheat straw 30% + bagasse 15% + wheat bran 15% + corn flour 10% and sawdust 30% + wheat straw 30% + rice bran 20% + soybean powder 10% + bagasse 10%, sawdust 60% + corn flour 40%, and sawdust 60% + rice bran 40%, with values of 90.34, 90.61, 90.13, and 90.07, respectively. Subsequently, at a concentration of 10 μg per milligram, the lowest inhibition in substrates sawdust 60% + bagasse 100%, corn flour 40%, sawdust 60% + Soybean powder 40%, and wheat straw 60% + rice bran 40%, with values of 1.87, 5.72, 7.35, and 8.04 μg/mg, respectively, was observed. Although a value of 90.61 was recorded in multicomponent substrates such as sawdust 30% + wheat straw 30% + rice bran 20% + soybean powder 10% + bagasse 10% substrate, the other substrates also showed high values. The synthetic antioxidant ascorbic acid had a higher inhibition activity percentage than the mushrooms harvested from 19 mixed substrates (Table 2).

In the experiment conducted by Angiolella et al. [48], the amount of free radical inhibition increased with increasing concentration, and the synthetic antioxidant BHA showed a higher free radical inhibition activity than the mushroom, which is consistent with the results of this research.

### 3.4. Cluster, PCA, and Pearson's Correlation Statistical Analysis

**Figure 3:**
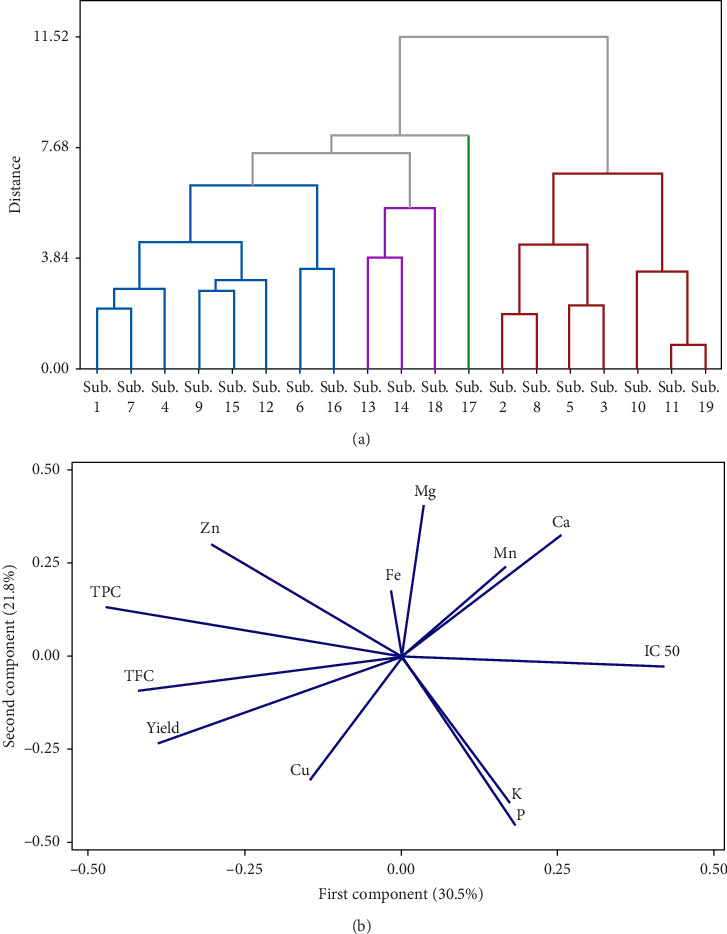
(a) Ward's cluster analysis of fruit bodies grew on 19 various substrates and (b) the principal component analysis (PCA) for mineral, mycochemical content, and antioxidant activity of fruit bodies grew on 19 various substrates.

The results of cluster analysis for different fruiting bodies from different substrates are shown in Figure 3. The cluster analysis (Figure 3(a)) identified four groups of substrates under the tested mushrooms that varied in terms of the mineral and mycochemical properties. Sub.13, Sub.14, Sub.18, and Sub.17 were placed in an independent group, and Sub.17 was grouped individually and separately. The findings also revealed that not only adding but also increasing the supplements into substrates led to the separation of substrates, revealing that the supplement caused more pronounced differences among the substrates. The PCA of minerals and mycochemical characteristics and antioxidant properties are shown in Figure 3(b). F1 and F2 represented 58.6% of the total variance, where F1 (30.5%) was driven by Mg, TFC, Mn, Ca, IC50, and K, P, and F2 (28.1%) were closely associated with Zn, TPC, and TFC. The contents of Mn, K, P, and Ca in fruit bodies were significantly negatively correlated with TPC and TFC. In contrast, Fe, Zn, and Mg had effects on the content of this element. The PCA reveals that the relative variance in the first component was affected by substrate enrichment (Figure 3(b)). However, there was no clear difference between the second component and relative variance among the treatments. TPC is closely related to the TFC variable. In addition, it can be seen that the grouping based on the PCA is partially consistent with the clustering results.

Under such conditions, fungi deliberately absorb large amounts of these elements from the substrate to supply their metabolic needs, especially when they are subjected to stressful conditions [49]. This finding corresponds to Nikkarinen and Mertanen [50] and Má et al. [51], who reported that the concentration relationship of the mineral elements between mushrooms and their substrate was already. However, in contrast to Malinowska et al. [52] and Chudzy´nski and Falandysz [53] did not find such a dependence.

The Pearson correlation analysis between minerals, antioxidant activity, and mycochemical contents of *H. erinaceus* harvested on different substrates is given in Table 3. About the mineral components with other traits, a positive linear correlation at the 0.01 level was obtained as follows: A positive linear correlation at the 0.05 level led to *r* = 0.383 for TFC and Ca, with *r* = 0.374 for TPC and Ca. A negative linear correlation was exhibited at the 0.05 level, with *r* = 0.45 for TPC and Zn and *r* = 0.468 for IC50 and Zn. Regarding the mycochemical component with other traits, a positive linear correlation was shown at the 0.01 level, *r* = 0.982 for TFC and TPC. A negative linear correlation was shown at the 0.05 level, *r* = 0.382 for yield and TFC, and *r* = 0.406 for yield and TPC. Based on the IC50, a positive linear correlation was shown at the 0.01 level, *r* = 0.973 for TPC and IC50, and *r* = 0.979 for TFC and IC50. These results are confirmed by Halliwell et al. [54] that phenolic components have a potential ability to scavenge free radicals and are considered an antioxidant content, which shows a positive correlation with other antioxidant activities. The presence of flavonoids and phenol in mushrooms can help to produce terpenoids and vitamins [40]. The current findings showed that phenolic and minerals compounds have a positive and significant correlation, which is confirmed by previous studies [55–58]. Mg and Ca had a strong positive correlation of *r* = 0.497 and *r* = 0.6, respectively, which is attributed to Kalac [28].

The findings of the present study revealed that there is a strong and positive correlation between phenolic compounds and IC50 (TFC, *r* = 0.979 and TPC, *r* = 0.973). The antioxidant activity is closely related to anthocyanin, flavonoids, vitamins, and TPC. However, based on many researchers' studies, the antioxidant activity is more influenced by total phenol [38]. Many other studies also reported that phenolic compounds obtained from mushroom extracts have showed excellent antioxidant activities by scavenging free radicals. These results can be indicated that these phenolic compounds may have more antioxidant power than other compounds identified in these mushrooms. This is consistent with the study of several authors who stated that the TPC correlated with the free RSA of other fungi [39, 40].

## 4. Conclusions

In this study, the potential of using selected agroindustrial wastes was studied to investigate their use as an alternative fruiting substrate for the cultivation of *H. erinaceus* as useful products. In conclusion, among the fruiting bodies' samples harvested from 19 substrates, the substrates sawdust 30% + wheat straw 30% + bagasse 15% + rice bran 15% + corn flour 10% recorded the highest phenol and flavonoid contents, with values of 27 mg GAE/g DW ext. and 8 mg QE/g DW ext. as well as the IC50 (88.7 μg/mL). P and K's highest amounts were recorded with the values of 1833 and 1600 mg/100 g DW, and Fe was recorded with values of 77.7 mg/100 g DW. This study, therefore, provides an understanding of biological technologies for the organic solid waste valorization into useful and valuable bio-based products with desirable medicinal aspects.

## Figures and Tables

**Table 1:** Micro- and macroelements in the *Hericium erinaceus* cultivated on the 19 different substrates.

Substrate content (%)	mg/100 g DW							
	Ca	Mg	K	P	Fe	Cu	Mn	Zn
Wheat straw 100	990 ± 36.10c	59.30 ± 3.06j	494 ± 5.29hij	69.70 ± 2.52i	23.30 ± 1.53ghi	0.53 ± 0.15cdefg	2.67 ± 0.58bc	2 ± 0efg
Sawdust 100	108 ± 2.89j	57.30 ± 2.52j	367 ± 2.65k	185 ± 5ghi	54 ± 1c	0.37 ± 0.06efgh	2.17 ± 0.76cd	3.67 ± 1.53bcdef
Bagasse 100	2267 ± 252.00a	1483 ± 76.40a	527 ± 25.20ghi	195 ± 5ghi	56.70 ± 2.89c	0.43 ± 0.21defgh	3.67 ± 0.58ac	4 ± 0bcdef
Wheat straw 60 + wheat bran 40	883 ± 15.30cd	145 ± 5.03efgh	633 ± 57.70efg	197 ± 11.50gh	18.70 ± 1.53ijk	0.40 ± 0.10defgh	4.33 ± 0.58b	4.67 ± 1.53abcd
Sawdust 60 + wheat bran 40	407 ± 11.50hi	171 ± 58.10ef	483 ± 15.30hij	303 ± 15.30efg	26 ± 1.73fg	0.73 ± 0.12abcde	1.57 ± 0.60cde	6 ± 1ab
Bagasse 60 + wheat bran 40	1270 ± 26.50b	237 ± 11.50cd	700 ± 0de	400 ± 50e	71.30 ± 2.31b	0.2 ± 0.05gh	0.90 ± 0.10def	1.67 ± 0.58fg
Wheat straw 60 + rice bran 40	1300 ± 100b	97.30 ± 2.52ghij	587 ± 15.30fgh	203 ± 5.77gh	9.83 ± 0.76lm	0.3 ± 0fgh	1.67 ± 0.58def	3 ± 2cdefg
Sawdust 60 + rice bran 40	473 ± 23.10gh	140 ± 10efgh	460 ± 17.30ijk	287 ± 41.60efg	24.70 ± 2.52gh	0.35 ± 0.09efgh	2.67 ± 0.58bc	4.33 ± 0.58abcde
Bagasse 60 + rice bran 40	1033 ± 57.70c	307 ± 40.40b	422 ± 13.10ijk	250 ± 50gh	46 ± 6.56 d	0.67 ± 0.35bcdef	2 ± 0cde	5 ± 2abc
Wheat straw 60 + corn flour 40	633 ± 57.70fg	80.00 ± 0ij	493 ± 60.30hij	147 ± 10.40hi	6 ± 1m	0.77 ± 0.31abcd	0.53 ± 0.06f	3.67 ± 1.15bcdef
Sawdust 60 + corn flour 40	345 ± 5hi	133 ± 5.77fghi	410 ± 26.50jk	282 ± 17.60efg	19.70 ± 1.15hij	0.97 ± 0.06ab	0.47 ± 0.06f	3.67 ± 0.58bcdef
Bagasse 60 + corn flour 40	800 ± 100de	193 ± 40.40de	360 ± 10k	390 ± 17.30ef	31.30 ± 1.15ef	0.63 ± 0.12bcdef	0.37 ± 0.23f	2.67 ± 0.58cdefg
Wheat straw 60 + soybean powder 40	1033 ± 57.70c	53.30 ± 5.77j	1000 ± 0c	1150 ± 50d	16.70 ± 3.51jk	0.10 ± 0h	2 ± 0cde	1.67 ± 1.15fg
Sawdust 60 + soybean powder 40	717 ± 28.90ef	135 ± 5.00efghi	1400 ± 100b	1277 ± 25.20bc	30.70 ± 2.52ef	0.87 ± 0.23abc	4.33 ± 0.58a	2 ± 0efg
Bagasse 60 + soybean powder 40	940 ± 36.10cd	92.30 ± 6.81ghij	760 ± 10d	1367 ± 57.70b	31.70 ± 0.58e	0.63 ± 0.31bcdef	2.33 ± 1.53bc	4 ± 1bcdef
Sawdust 30 + wheat straw 30 + 15 bagasse + rice bran 15 + corn flour 10	257 ± 40.40ij	89 ± 1hij	683 ± 15.30def	1167 ± 153cd	77.70 ± 2.52a	0.77 ± 0.06abcd	0.39 ± 0.28f	2.33 ± 0.58defg
Sawdust 30 + wheat straw 30 + bagasse 15 + wheat bran 15 + corn flour10	640 ± 36.10ef	267 ± 28.90bc	677 ± 20.80def	390 ± 26.50ef	32.30 ± 0.58e	0.60 ± 0.26bcdef	2 ± 1cde	6.67 ± 2.08a
Sawdust 30 + wheat straw 30 + rice bran 20 + soybean powder 10 + bagasse 10	1017 ± 28.90c	142 ± 7.64efgh	1600 ± 100a	1833 ± 153a	14 ± 1kl	0.90 ± 0.17abc	0.85 ± 0.74def	0.60 ± 0.1g
Sawdust 30 + wheat straw 30 + wheat bran 20 + soybean powder 10 + bagasse 10	357 ± 11.50hi	152 ± 7.64efg	423 ± 20.80ijk	273 ± 2.65fg	10.30 ± 3.06lm	1.07 ± 0.12a	0.77 ± 0.06ef	3 ± 1cdefg

*Note:* Average values (three replicates) ± standard error are given. Means followed in a column with the same letter are not significantly different by LSD at the 0.01 probability level.

**Table 2:** DPPH free radical scavenging activity of fruiting bodies harvested from 19 different substrates in five different concentrations.

Substrate content (%)	Concentration (μg/mL)				
	10	30	50	100	200
Wheat straw 100	22.277 ± 0.63dC	46.713 ± 4.22efB	87.043 ± 2.62cA	85.667 ± 0.58deA	85.647 ± 1.12efA
Sawdust 100	13.747 ± 1.56ghC	44.723 ± 0.63fB	77.927 ± 0.89efA	77.7 ± 0.61hA	78.26 ± 1.1iA
Bagasse 100	5.72 ± 0.63kC	31.147 ± 1.23lB	74.84 ± 0.77gA	74.917 ± 1.66hA	75.93 ± 0.9iA
Wheat straw 60 + wheat bran 40	18.16 ± 0.28efE	34.71 ± 0.62ijkB	64.917 ± 0.14hC	75.9 ± 0.85hB	77.983 ± 0.98iA
Sawdust 60 + wheat bran 40	18.187 ± 0.32efD	47.143 ± 1.22efC	79.807 ± 0.73deB	85.673 ± 0.58deA	85.92 ± 1.59defA
Bagasse 60 + wheat bran 40	21.277 ± 1.11dE	40.2 ± 1.06ghD	66.047±1hC	75.067 ± 1.01hB	78.26 ± 1.1iA
Wheat straw 60 + rice bran 40	8.04 ± 1jkD	33.083 ± 1.01klC	60.14 ± 1.03iB	82 ± 1fgA	83.227 ± 1.07fghA
Sawdust 60 + rice bran 40	19.733 ± 1.55deD	48.863 ± 1.03deC	80.743 ± 0.65dB	89.167 ± 1.04bcA	90.07 ± 1.01bcA
Bagasse 60 + rice bran 40	10.033 ± 0.95ijD	34.077 ± 1.01jklC	61.217 ± 1.07iB	80.867 ± 0.78gA	82.287 ± 0.5hA
Wheat straw 60 + corn flour 40	18.16 ± 1.04efC	35.043 ± 1ijkB	75.78 ± 0.7fgA	77.667 ± 0.58hA	78.323 ± 1.53iA
Sawdust 60 + corn flour 40	1.87 ± 1.2lD	38.927 ± 1.67ghC	85.167 ± 0.76cB	87.833 ± 0.76cdA	90.13 ± 0.81bcA
Bagasse 60 + corn flour 40	11.353 ± 1.52hiC	37.66 ± 0.57ghiB	75.78 ± 0.7fgA	76.7 ± 0.61hA	78.05 ± 0.93iA
Wheat straw 60 + soybean powder 40	17.357 ± 0.62efC	36.993 ± 0.99hijB	80.473 ± 0.5dA	80.833 ± 1.44gA	82.62 ± 1.51ghA
Sawdust 60 + soybean powder 40	7.3533 ± 0.56jkE	32.257 ± 0.25klD	55.457 ± 0.08jC	84.667 ± 1.53efB	88.527 ± 1.5bcdA
Bagasse 60 + soybean powder 40	15.48 ± 0.83fgD	40.803 ± 0.73gC	67.343 ± 0.57hB	76 ± 2hA	76.113 ± 0.2iA
Sawdust 30 + wheat straw 30 + bagasse 15 + rice bran 15 +corn flour 10	8.9033 ± 1.15ijC	50.59 ± 1.02cdB	86.377 ± 1.52cA	85.833 ± 1.61deA	87.587 ± 2.5cdeA
Sawdust 30 + wheat straw 30 + bagasse 15 + wheat bran 15 + corn flour10	30.697 ± 2.13bC	56.25 ± 1.09abB	90.403 ± 2.51bA	90.767 ± 2.54bA	90.34 ± 0.59bcA
Sawdust 30 + wheat straw 30 + rice bran 20 + soybean powder 10 + bagasse 10	27.587 ± 3.17cC	53.117 ± 2.89bcB	90.13 ± 0.81bA	89.683 ± 1.53bcA	90.613 ± 1.06bA
Sawdust 30 + wheat straw 30 + wheat bran 20 + soybean powder 10 + bagasse 10	8.1267 ± 1.63jkC	40.803 ± 0.73gB	81.683 ± 1.53dA	83.867 ± 1.63efA	85.357 ± 2.51efgA
Ascorbic acid	39.438 ± 2.14aC	59.61 ± 2.26abB	93.157 ± 0.27aA	94.167 ± 0.29aA	94.43 ± 0.51aA

*Note:* Values were considered as mean ± SD of three replicates. A–D: values with various superscripts in the same row are significantly different at the level of 1% (*p* < 0.01), a–l: values bearing the different superscript in the same column are significantly different at the level of 1% (*p* < 0.01).

**Table 3:** Correlations analysis between mineral, chemical, phenolic content, and antioxidant activity of *Hericium erinaceus* fruiting body.

	Ca	Mg	K	P	Fe	Cu	Mn	Zn	TPC	TFC	IC50
Yield	−0.316	−0.283	0.463⁣^∗^	0.646⁣^∗∗^	−0.179	0.502⁣^∗∗^	−0.282	0.017	−0.406⁣^∗^	−0.382⁣^∗^	−0.357
TPC	0.374⁣^∗^	0.023	−0.097	−0.146	−0.248	−0.41	0.15	−0.45⁣^∗^	1	0.982⁣^∗∗^	0.973⁣^∗∗^
TFC	0.383⁣^∗^	0.013	−0.062	−0.092	−0.176	−0.429	0.106	−0.518	0.982⁣^∗∗^	1	0.979⁣^∗∗^
IC50	0.361	0.021	−0.079	−0.096	−0.215	−0.418	0.091	−0.468⁣^∗^	0.973⁣^∗∗^	0.979⁣^∗∗^	1

⁣^∗^Significant at the 0.05 level (2-tailed).

⁣^∗∗^Significant at the 0.01 level (2-tailed).

## Data Availability

The data used to support the findings of this study are available on reasonable request to the corresponding author.
